# Dysfunctional adipose tissue and low-grade inflammation in the management of the metabolic syndrome: current practices and future advances

**DOI:** 10.12688/f1000research.8971.1

**Published:** 2016-10-13

**Authors:** Marleen M. J. van Greevenbroek, Casper G. Schalkwijk, Coen D.A. Stehouwer

**Affiliations:** 1 Maastricht University Medical Center, Maastricht, 6229 ER, Netherlands; 2 CARIM School for Cardiovascular Diseases, Maastricht, 6229 ER, Netherlands; 3 Academic Hospital Maastricht, Maastricht, 6229 HX, Netherlands

**Keywords:** metabolic syndrome, adipose tissue dysfunction, low-grade inflammation, inflammasome

## Abstract

The ongoing worldwide obesity epidemic makes the metabolic syndrome an increasingly important entity. In this review, we provide a short background on the metabolic syndrome, we discuss recent developments in the three main options that have been identified for intervention in the metabolic syndrome, i.e. lifestyle and surgical and pharmacological interventions, and we focus on different views in the literature and also include our own viewpoints on the metabolic syndrome. In addition, we discuss some emerging treatment targets for adipose tissue dysfunction and low-grade inflammation, i.e. activation of the inflammasome and the complement system, and consider some selected opportunities for intervention in these processes.

## The metabolic syndrome

In 1988, Reaven described a constellation of related variables that tend to occur concurrently in an individual. It was anticipated to be of relevance for the development of coronary artery disease ^1^. He referred to this constellation as “Syndrome X”. Key components of Syndrome X were identified as (a) resistance to insulin-stimulated glucose uptake, which is reflected by, for example, glucose intolerance and hyperinsulinemia, (b) dyslipidemia that is characterized by increased very-low-density lipoprotein triglyceride and decreased high-density lipoprotein (HDL) cholesterol, and (c) hypertension. Reaven postulated that insulin resistance was the key common feature of Syndrome X, with other observed metabolic abnormalities deriving from this overall underlying cause ^1^.

The relevance of Syndrome X for understanding cardiometabolic health has been retained since its first description, although the syndrome is nowadays mainly referred to as “the metabolic syndrome”. The concept and definition of the metabolic syndrome have evolved over time, and the most widely accepted current definition of the metabolic syndrome is three abnormal findings out of the following components: raised blood pressure, dyslipidemia (raised triglycerides and/or lowered HDL cholesterol), raised fasting glucose, and central obesity. For each component, a single (if relevant, sex-specific) cut point is used, except central obesity for which national or regional cut points for waist circumference can be used ^2^. So, in contrast to the original description of Syndrome X, the goal for the metabolic syndrome is to apply a set of easily obtainable measures to identify individuals who have a relatively high risk of developing cardiovascular disease (CVD) and type 2 diabetes mellitus (T2DM). Notably, individuals who have the metabolic syndrome will be more insulin resistant than those who do not, which is in line with the original concept that was proposed by Reaven ^1^.

**Figure 1.**
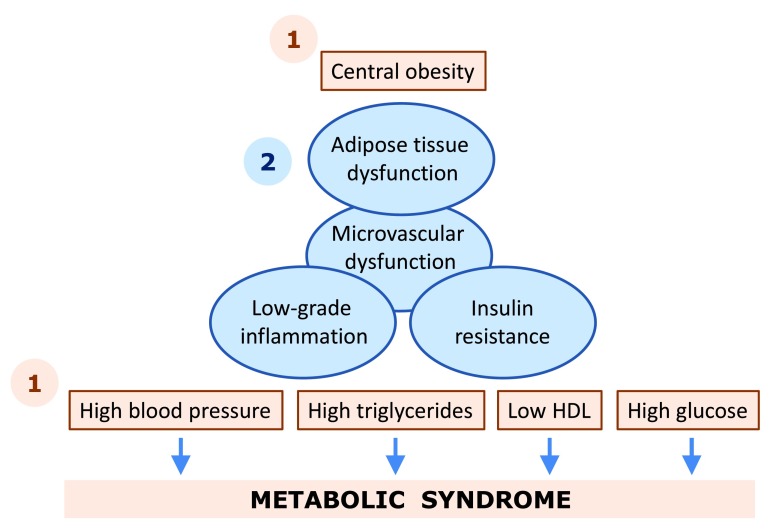
Schematic representation of the metabolic syndrome. The components that are used to define the metabolic syndrome are indicated in light brown. The underlying metabolic derangements are indicated in blue. Interventions can be targeted at the level of one or multiple individual components (indicated with 1) or at the potential underlying causes of the metabolic syndrome (indicated with 2). We have proposed that microvascular dysfunction is an early functional pathway that connects adipose tissue dysfunction to insulin resistance and resulting metabolic derangements, but this is beyond the scope of this review ^9^. Abbreviations: HDL, high-density lipoprotein.

Individuals who have the metabolic syndrome are also more likely to be in a state of ongoing generalized low-grade inflammation. An emerging view of the metabolic derangements that underlie the metabolic syndrome is that dysfunctional adipose tissue drives the development of low-grade inflammation and insulin resistance ^2– 4^. Within that view, low-grade inflammation can, in the causal path towards the development of the metabolic syndrome, be placed downstream of obesity and upstream of insulin resistance and the metabolic hallmarks of the metabolic syndrome, i.e. dyslipidemia, hyperglycemia, and hypertension ^5^. A possible alternative concept is that dysfunctional adipose tissue can induce the metabolic derangements associated with the metabolic syndrome via other pathways. For instance, increased release of fatty acids can induce insulin resistance ^6^ and high triglycerides/low HDL ^7^ as well as an inflammatory response ^8^. One of the earliest functional derangements that can be seen in the metabolic syndrome is microvascular dysfunction. We and others have proposed that obesity-induced microvascular dysfunction (including functional [e.g. impaired endothelial function of different microvascular beds and/or diminished capillary recruitment] as well as structural [e.g. decreased capillary density and/or structural remodeling] impairments) may also be a relevant cause for insulin resistance and diabetes in individuals with the metabolic syndrome ^9, 10^ ( Figure 1).

The metabolic syndrome is of growing relevance for public health, not only in the general population but also in specific subpopulations that are more likely to have, or to develop, the syndrome. For example, human immunodeficiency virus (HIV)-infected individuals on highly active anti-retroviral therapy (HAART) were among the first who were identified as a high-risk subgroup ^11^. Nowadays, the focus is also on elderly individuals ^12^, for example, not just for the development of cardiometabolic diseases (including CVD and T2DM) but also for functional ^13^ and potentially cognitive ^14– 16^ disability related to the metabolic syndrome. Also, optimal management of the metabolic syndrome in these individuals may differ from that in the general population, among other reasons because of the higher prevalence of sarcopenic obesity in the elderly ^17^. Moreover, the presence of the metabolic syndrome as a comorbid condition may adversely affect (or be affected by) the course and treatment of an underlying disease such as psoriasis, sleep apnea, mental and cognitive diseases, and chronic obstructive lung disease ^14, 18– 22^.

## The metabolic syndrome in research and care

Over the past 30 years, the metabolic syndrome has been accepted as a relevant health determinant. Notwithstanding this general view, there is debate on whether or not the metabolic syndrome should be regarded as an actual disease. In 2011, more than 20 years after his seminal publication on Syndrome X, Reaven published a position paper in which he challenged the usefulness of the metabolic syndrome as a diagnostic category ^23^. His main conclusion was that, within a population of apparently healthy individuals, the metabolic syndrome appeared to be less effective than the Framingham Risk Score in predicting CVD and no better than fasting plasma glucose concentrations in predicting T2DM. Similar views have been expressed by others ^24^. This suggests that the clinical utility of the metabolic syndrome as a diagnostic tool may be limited. Despite this limitation of the metabolic syndrome as a risk indicator for those specific diseases, individuals with the metabolic syndrome are generally characterized by central obesity accompanied by generalized low-grade inflammation (i.e. higher plasma concentrations of C-reactive protein, interleukin [IL]-6, and other inflammatory markers) and insulin resistance and do have a higher chance of developing CVD and/or T2DM ^2, 25– 27^. The think tank of the CardioMetabolic Health Alliance recently proposed that the metabolic syndrome “is a complex pathophysiological state comprised of a cluster of clinically measured and typically unmeasured risk factors, is progressive in its course, and is associated with serious and extensive comorbidity, but tends to be clinically under-recognized” ^28^. Recommendations for the management of the metabolic syndrome were focused on therapeutic lifestyle changes, including a healthy diet and regular physical activity, to address all aspects of the metabolic syndrome and, in addition, to treat individual components using specific interventions. A key recommendation was that “a new care model for patients with MetS [metabolic syndrome] is essential and should include screening, risk stratification, and algorithmic management of patients according to the specific subtype and stage” ^28^. Thus, subphenotyping in order to provide tailored care for individual subtypes of the metabolic syndrome was advised.

In summary, the metabolic syndrome is a very useful concept for both clinicians and researchers. Although its applicability as a tool for risk prediction may be limited, it may be used as an additional tool to guide treatment. An important advantage of the metabolic syndrome is that it comprises a very useful concept to evaluate and understand the biology that underlies cardiometabolic diseases and, as such, to identify novel targets for drugs to treat and prevent those diseases. It is, however, a rather crude tool and its use both for the treatment of affected individuals and in research to understand the underlying biology and identify novel treatment targets may benefit from further fine-tuning and subphenotyping of the metabolic syndrome.

## Current developments in the management and treatment of the metabolic syndrome

The metabolic syndrome can be managed via several strategies. The first class of interventions in the metabolic syndrome aims at adopting a healthy lifestyle. Initially, such lifestyle interventions were aimed primarily at weight reduction through dietary and physical activity interventions. Virtually every lifestyle intervention that successfully addresses obesity in a population with the metabolic syndrome will simultaneously improve metabolic characteristics. However, time and again it has been shown that such major lifestyle changes are hard to maintain.

More recently, alternative lifestyle interventions have been employed that target the quality of the diet and the fine-tuning of physical activity advice. Specific dietary components have obtained attention. For instance, the consumption of tree nuts has, in a meta-analysis of randomized controlled studies, been shown to lower triglycerides and fasting blood glucose with no effects on waist circumference, HDL cholesterol, or blood pressure ^29^, and higher dairy consumption has, in a meta-analysis of observational studies, been shown to be associated with less frequent occurrence of the metabolic syndrome ^30^. The health effects of such dietary adaptations may seem subtle, but at the population level their effects may be substantial and such changes in dietary habits may be more achievable than, for example, rigorous caloric restriction. Similar developments have occurred in physical activity interventions. Initially, the focus was on training interventions such as low and high aerobic and resistance training methods. Nowadays, alternative strategies such as limiting of, and increasing breaks in, sedentary behavior ^31, 32^ and also alternative physical activity interventions such as yoga ^33, 34^ have been reported to have beneficial effects on several aspects of the metabolic syndrome.

A second class of possible interventions for the metabolic syndrome is targeted specifically toward the obesity component of the metabolic syndrome. This class comprises the growing range of surgical interventions that are available. This type of intervention is indicated only for individuals with the metabolic syndrome who have a body mass index (BMI) of more than ~35–40 kg/m ^2^. In general, bariatric procedures will result in rapid improvement of several metabolic variables and substantial weight loss ^35^. Unfortunately, only a limited number of bariatric intervention trials have reported high-quality (i.e. less than 20% attrition), long-term (>5 years) follow-up data ^36^. A recent meta-analysis with a median follow-up time of 3 years (interquartile range 24–48 months) reported that a median decrease in BMI of ~12–14 kg/m ^2^ was associated with a 3-fold reduction in T2DM and hyperlipidemia and 2-fold reduction in hypertension by the end of the follow-up period ^37^. Currently, the most-used procedures include adjustable gastric banding, vertical sleeve gastrectomy, Roux-en-Y gastric bypass, and biliopancreatic diversion with duodenal switch ^38^. Novel developments in surgical interventions focus on less invasive techniques that mimic the approach of the existing interventions ^39^.

The third class of interventions in the metabolic syndrome comprises pharmacological treatment options. Often such pharmacological interventions target individual components of the metabolic syndrome, i.e. obesity, hypertension, dyslipidemia, and hyperglycemia ( Figure 1). Both classic and novel targets can be addressed with existing and novel drugs. In addition to managing the components that define the metabolic syndrome, pharmacological interventions in the metabolic syndrome also aim to address the possible underlying pathology, i.e. dysfunctional adipose tissue, low-grade inflammation, and insulin resistance ( Figure 1). One classic compound that has been on the market for >20 years now is metformin. Metformin is the first choice of glucose-lowering drug in obesity-associated diabetes and can also prevent the progression from prediabetes to diabetes ^40^; it stimulates AMP-activated protein kinase (AMPK) activity, which leads to a reduction in hepatic glucose production and improvement of insulin sensitivity and may additionally have anti-inflammatory effects ^41^ and improve endothelial function ^42^. For these reasons, metformin has been put forward as a potentially powerful tool to control the adverse health consequences of the metabolic syndrome ^43, 44^.

In this narrative review, we will highlight some exciting emerging targets for novel compounds to manage the potential primary metabolic aberrations in the metabolic syndrome, i.e. adipose tissue dysfunction and low-grade inflammation.

## Novel targets in the metabolic syndrome: managing dysfunctional adipose tissue and low-grade inflammation

**Figure 2.**
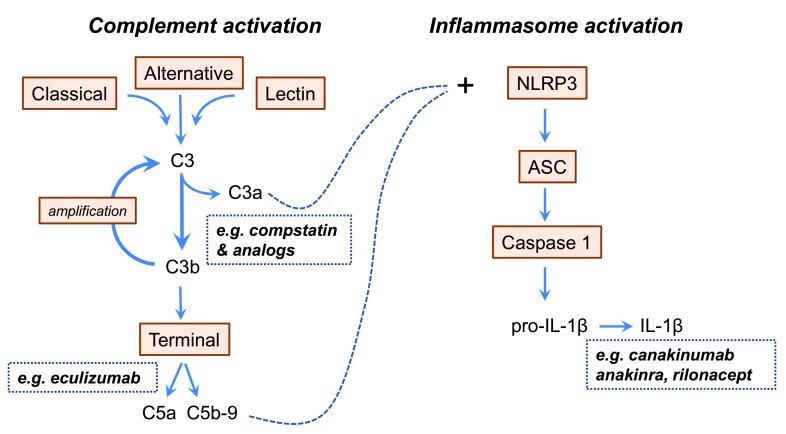
Relevant aspects of complement activation and inflammasome activation with their possible interconnection. The inflammasome and the complement system can be activated via so-called danger-associated molecular patterns (DAMPS) and pathogen-associated molecular patterns (PAMPS). Activation of the classical, the lectin, and the alternative pathway of complement activation all result in the activation of complement C3. The alternative pathway functions as an amplification route for all pathways of complement activation. Activation of C3 leads to the generation of C3a and the activation of the terminal effector pathway. Activation of the NOD-like receptor family Pyrin domains-containing protein 3 (NLRP3) inflammasome includes the formation of a cytosolic multiprotein complex that activates caspase 1. Caspase 1 cleaves pro-interleukin (IL)-1β into IL-1β. Pharmacological compounds that are used to dampen the effects of inflammasome activation primarily act at the level of IL-1β. In the dotted squares, we have indicated some selected drugs that are already used to target complement and/or inflammasome activation in other diseases. Compstatin inhibits complement activation by binding C3 and interfering with convertase formation and C3 cleavage ^90^; eculizumab binds to complement protein C5, thus inhibiting the formation of C5a and the terminal complement complex C5b-9 ^85^. Canakinumab (a neutralizing monoclonal anti-IL-1β), anakinra (an IL-1 receptor antagonist), and rilonacept (a soluble decoy IL-1 receptor) all block the IL-1β effector pathways of inflammasome activation ^56^. The intermittent lines indicate the emerging link between activated complement and the NLRP3 inflammasome ^91^. Abbreviations: ASC, apoptosis-associated speck-like protein containing a CARD.

As indicated in the previous paragraphs, dysfunctional adipose tissue is considered one of the primary origins of the metabolic disturbances that are present in the metabolic syndrome. Novel intervention strategies that aim to improve adipose tissue function and health may therefore add to the resolution of the most basic problem in the metabolic syndrome. In recent years, several pharmacological interventions aimed at specific inflammatory pathways have already been explored as treatment targets for T2DM. So far, the effects of these interventions have been modest, with limited effects on insulin resistance, hyperglycemia, and some aspects of systemic inflammation ^45^. These modest effects might reflect the causal role of inflammation in T2DM and possibly the metabolic syndrome is limited, but on the other hand it may reflect that the optimal treatment target(s) and strategy have not yet been identified ^45^. Here, we will focus on the inflammasome and the complement system as two potential contributors to adipose tissue dysfunction ( Figure 2). The inflammasome is an important cellular danger-sensing system that has been proposed to be the central regulator of early adipose tissue inflammation ^46^; the complement system is a key component of innate immune defense against infection and also plays roles in the regulation of cell and tissue homeostasis ^47, 48^. Complement is increasingly recognized as a relevant player in adipose tissue function and metabolism ^49– 51^.

The NOD-like receptor family Pyrin domains-containing protein 3 (NLRP3) is one of the best characterized members of the inflammasome family ^52^. NLRP3 may be causally involved in the induction of obesity and insulin resistance ^46, 53^, at least in mouse models. Several endogenous stress signals such as reactive oxygen species, glucose, and palmitate may act as *in vivo* inducers of the inflammasome ^52^. IL-1β is the main inflammatory mediator that is induced via NLRP3 inflammasome activation and is known for its central role in inflammatory processes ^54^. The NLRP3 inflammasome thus conceptually provides an attractive target in the control of obesity-associated chronic inflammation. Design of drugs that modulate and control inflammasome activation and IL-1β activity is an area of active research. The treatment option that is closest to clinical application is to target IL-1β and thereby inhibit the main effector pathway of inflammasome activation ^55, 56^. IL-1β activity can be blocked via, for example, IL-1 receptor antagonists or anti IL-1β antibodies ( Figure 2). These compounds have already been used in patients with T2DM ^57^ and rheumatologic diseases ^58^ and have, until now, generally been reported to be safe ^59^. Blocking the IL-1β pathway resulted in less inflammation in patients with T2DM accompanied by improved glycemic control and less insulin use, which appeared to be due to improved β-cell function ^56, 60^.

The complement system is a complex protein network that was initially identified as part of the innate immune system, but it also plays a relevant role in cell and tissue homeostasis ^48^. The complement system has been implicated in cardiometabolic diseases, including the metabolic syndrome ^47, 61, 62^. Complement activation is strongly associated with low-grade inflammation in the metabolic syndrome and related cardiometabolic diseases ^63– 70^. Notably, we have shown in observational studies in humans that activated complement factors are strongly associated with markers of endothelial dysfunction ^64, 65^, which are thought to reflect mainly greater microvascular endothelial dysfunction ^10^. In particular, the plasma concentration of complement C3, the central complement component, has been implicated in the development and pathogenesis of the metabolic syndrome ^71– 74^. In addition, plasma C3 concentration has been shown to be an independent risk factor for T2DM ^75– 77^. The association between complement C3 and incident CVD is somewhat less consistent. C3 was shown to be a risk factor for CVD ^77– 79^ although not always independent of covariates ^78^. In addition, properdin, a positive regulator of C3 activation, showed a positive longitudinal association with incident cardiovascular events ^64^. The activation of C3 leads to the generation of the anaphylatoxin C3a and the opsonin C3b ^48^. In animal models, signaling via the C3–C3a–C3a-receptor axis has been shown to modulate insulin resistance and adipose tissue inflammation ^69, 80^. Other aspects of the complement system with potential relevance in adipose-tissue-mediated inflammation and insulin resistance include the terminal pathway of complement activation, which leads to the generation of the proinflammatory anaphylatoxin C5a and the (sub)lytic terminal complement complex (TCC, also known as C5b-9). In human cohorts and in animal studies, C5a and TCC were associated with low-grade inflammation ^65, 70, 81^. Notably, the terminal pathway of complement activation has been implicated in the activation of the NLRP3 inflammasome ^82– 84^. These data suggest that control of complement activation may be instrumental in the control and/or prevention of the metabolic syndrome. Some of these compounds have already been used in patients with different pathologies ranging from severe diseases of complement dysregulation such as atypical hemolytic uremic syndrome (aHUS) via diseases with local complement activation and/or infection such as age-related macular degeneration and periodontitis to chronic inflammatory diseases such as rheumatoid arthritis ^85– 88^. Damage to endothelial cells is a primary effect of the uncontrolled activation of the alternative complement pathway that is characteristic for aHUS. This results in thrombotic microangiopathy with severe end-organ damage, usually to the kidneys. The anti-C5 monoclonal antibody eculizumab was shown to effectively inhibit complement-mediated thrombotic microangiopathy and improve renal function ^85^. Local inhibition of complement at the level of C3 activation in age-related macular degeneration resulted in less local inflammation ^86^. In a non-human primate model of periodontitis, Cp40, a compstatin analog, prevented tissue damage and resulted in lower local concentration of inflammatory effectors, including IL-1β ^87^. In patients with rheumatoid arthritis, eculizumab resulted in modest improvement in disease activity, which was accompanied by reduction in C-reactive protein concentration ^88^. In summary, human data on pharmacological agents that modulate low-grade inflammation via complement inhibition are currently scarce, but we hypothesize that the control of complement activation may prove to be a useful early target to control obesity-induced low-grade inflammation in the metabolic syndrome. The development of novel drugs to control complement activation is a rapidly evolving research field ^89^.

## Future perspectives for the management of the metabolic syndrome

In summary, the metabolic syndrome is characterized by obesity, hypertension, dyslipidemia, and/or hyperglycemia, and each of these characteristics can be addressed to improve the health status of an individual with the metabolic syndrome. The metabolic syndrome is also characterized by dysfunctional adipose tissue and low-grade inflammation, and addressing these basic aspects of the metabolic syndrome can have major beneficial effects on the prevention and regression/resolution of the metabolic syndrome. There are various options to address dysfunctional adipose tissue and low-grade inflammation. A healthy lifestyle characterized by moderate and varied dietary habits and moderate physical activity accompanied by a normal weight is one of the most efficient ways to maintain healthy adipose tissue depots with no-to-minimal occurrence of inflammation. Thus, regardless of emerging pharmacological treatment options, lifestyle interventions should remain the primary intervention for individuals with the metabolic syndrome. Notwithstanding, we acknowledge and emphasize that long-lasting lifestyle changes are often not achieved. Hence, pharmacological compounds that target the core of the metabolic syndrome, i.e. the dysfunctional fat depot accompanied by generalized low-grade inflammation, are still needed. The control of inflammasome and complement system activation, for example, may comprise such targets.
